# Correlative volume microcopy of virus-containing amphisomes enabled by fluorescence-guided focused ion beam-scanning electron microscopy

**DOI:** 10.1016/j.xpro.2025.104020

**Published:** 2025-08-15

**Authors:** Miriam Susanna Lucas, Katarina Wendy Schmidt, Christian Münz

**Affiliations:** 1 Scientific Center for Optical and Electron Microscopy (ScopeM), ETH Zurich, 8093 Zurich, Switzerland; 2 Viral Immunobiology, Institute of Experimental Immunology, University of Zurich, 8057 Zurich, Switzerland

**Keywords:** Cell-based Assays, Immunology, Microscopy

## Abstract

Volume correlative light and electron microscopy (vCLEM) enables ultrastructural analysis of rare cellular events. Here, we present a protocol for targeting virus-containing amphisomes in eukaryotic cells with vCLEM. We describe steps for identifying target cells using confocal fluorescence microscopy, preparing and mounting samples for focused ion beam-scanning electron microscopy (FIB-SEM), relocating the target cells and FIB-SEM stack acquisition, and correlation of volume light and electron microscopy data for 3D modeling. This protocol is optimized for imaging adherent cultured cells.

For complete details on the use and execution of this protocol, please refer to Schmidt et al.^1^

## Highlights

• A vCLEM protocol for fluorescence-guided FIB-SEM of rare cellular events
• Instructions for using gridded glass-bottom dishes to relocate cells of interest
• Sample preparation steps for staining, dehydration, and resin embedding for FIB-SEM
• Workflow for using mitochondria as internal landmarks for high-accuracy correlation

## Abstract (editor highlights)

Publisher’s note: Undertaking any experimental protocol requires adherence to local institutional guidelines for laboratory safety and ethics.

## Graphical abstract

**Figure:**
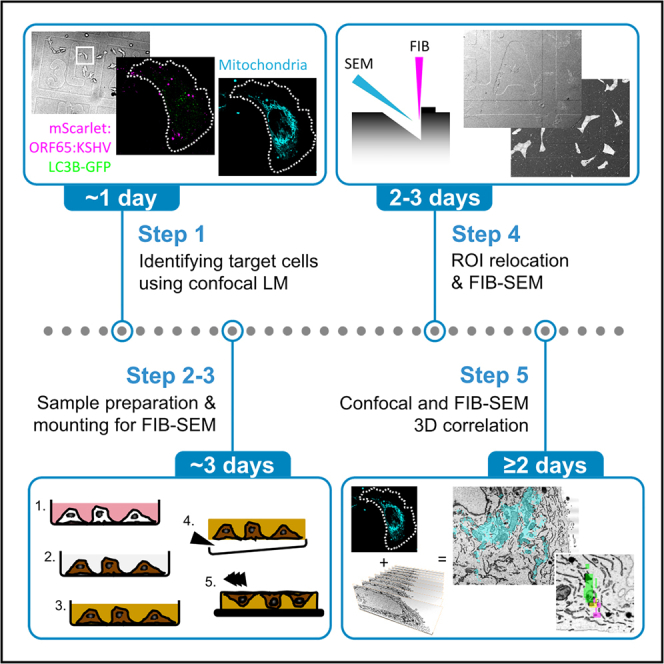

## Before you begin

Kaposi sarcoma-associated herpesvirus (KSHV) is an oncogenic γ-herpesvirus. LC3B (cytosolic microtubule-associated protein light chain 3B) is a key protein involved in the process of macroautophagy that delivers cytoplasmic constituents to lysosomal degradation. During KSHV infection, LC3B was found to co-localize with KSHV in amphisomes, indicating that autophagosomes fuse with virus-containing endosomes. Down-regulation of autophagy-related (ATG) proteins increased KSHV infection, suggesting that macroautophagy acts as a protective immune mechanism against this virus. Using a recombinant virus expressing mScarlet coupled to the small virus capsid protein encoded by ORF65, we tracked KSHV infection in U2OS osteosarcoma cells. Autophagosomes were identified via GFP-tagged LC3B which gets covalently attached to autophagosomal membranes. Since in confocal data, only about 15% of KSHV mScarlet puncta co-localized with GFP-LC3B an orchestrated correlative approach was indispensable.

Cells are grown in glass-bottom dishes with a gridded coverslip containing an alphanumeric pattern, and areas of co-localization between the viral capsid and LC3B are identified through confocal immunofluorescence microscopy. Subsequently, samples are stained with heavy metal salts, dehydrated, and embedded in resin. After detaching the glass coverslip from the polymerized resin, the surface features of the gridded coverslip are imprinted onto the resin surface, making it visible as a topographical pattern on the resin. This imprinted pattern is used as a reference for relocating the cells of interest in FIB-SEM.^2^^,^^3^

Instead of adding artificial fiducial markers, which could interfere with cellular integrity or interact with the virus internalization procedure, we used mitochondria labeled with MitoTracker Deep Red as internal fiducials. Mitochondria are abundant and well-defined in both confocal and FIB-SEM images. Their distinctive morphology makes them a reliable reference point for image alignment and correlation.

In addition to studying virus entry, this protocol can be applied to investigate dynamic cellular events such as vesicle trafficking, protein localization, and organelle ultrastructure, where both the functional aspects (observed through fluorescence microscopy) and structural details (revealed by electron microscopy) are necessary for a comprehensive understanding. Any fluorescent label to identify the target of interest may be used in this protocol. This includes genetically encoded tags such as GFP or mCherry, as well as synthetic fluorophores like Alexa Fluor dyes, cyanine-based fluorophores, or quantum dots. However, it is important that the chosen label does not require the use of detergents, as these may alter ultrastructure. Additionally, the retention of fluorescence after chemical fixation should be confirmed. This flexibility makes the protocol broadly applicable to a wide range of research questions in cell biology and virology.

### Institutional permissions

The Laboratory of Viral Immunobiology at the Institute of Experimental Immunology investigates recombinant KSHV viruses (KSHV.219) in gene modified cell cultures with the permission of the Federal Office of Public Health of the Swiss Confederation (BAG) under approval number A081009.

### Cell culture, virus production, and virus infection

**Timing: 5–6 days**

The following section outlines the production of the specific virus strain and the infection conditions used in the experiment described by Schmidt et al.^1^ These conditions may vary depending on the cell type and virus strain used. Nonetheless, the CLEM protocol described below is compatible with other light microscopy-based cell assays, provided the cells are cultured on gridded coverslips.

1. Virus production and titration.
  a. Produce mScarletH:ORF65.KSHV^4^ from BAC16 bacmid iSLK cells,^5^ which are grown in Dulbecco’s modified Eagle medium (DMEM) supplemented with 10% fetal bovine serum with 200 mg/mL hygromycin B, 250 mg/mL geneticin, and 2.5 mg/mL puromycin.
  b. For lytic cycle induction, remove the selection drugs and add 1 mg/mL doxycycline and 2.5 mmol sodium butyrate.
  c. After 4 days of incubation at 37°C, harvest the supernatant, discard the producer iSLK cells, and filter the virus containing supernatant through 1.2 μm sterile syringe filters.
  d. Concentrate the mScarletH:ORF65.KSHV-containing supernatant by ultracentrifugation at 30′000 × *g* at 4°C for 2 h.
  e. Resuspend the viral pellet gently in 50–100 μL DMEM on ice, as not to damage the virions.
  f. To determine the titers of the produced mScarletH:ORF65.KSHV, make several dilutions and infect 50′000 U2OS cells seeded on Ø 12 mm round glass coverslips in a 24-well plate for 60 min.
  g. Prepare PBS from 2.68mmol/L potassium chloride, 8mmol/L disodium hydrogen phosphate, 1.8mmol/L potassium dihydrogen phosphate and 136.9mmol/L sodium chloride.
  h. Wash the coverslips in PBS to remove unattached virions and then fix on the coverslips in 4% formaldehyde for 5 min.
  i. After washing again, mount cells using Dako mounting medium (Agilent) in presence of 1 mg/mL of DAPI.
  j. Analyze by confocal light microscopy (excitation max. 569 nm, emission max. 594 nm) to determine virus concentrates by immunofluorescence analysis of the average number of mScarletH-ORF65 puncta per U2OS cell.

***Note:*** This number is used to determine the multiplicity of infection (MOI).

2. Culture target cells in 35 mm gridded glass bottom dishes.
  a. Culture GFP-LC3B transduced U2OS osteosarcoma cells in DMEM, supplemented with 10% fetal bovine serum, 1% L-Glutamine, 50 U/mL penicillin-streptomycin for 24–48 h to allow proper attachment to the glass substrate.
  b. Grow the cells in a 5% CO_2_ atmosphere at 37°C.
3. Virus infection.
  a. Infect GFP-LC3B transduced U2OS cells with mScarletH:ORF65.KSHV with a MOI of 10 for 30 min.

## Key resources table

**Table:** 

REAGENT or RESOURCE	SOURCE	IDENTIFIER
**Bacterial and virus strains**		

mScarletH:ORF65.KSHV	Schlagowski et al.^4^	N/A

**Chemicals, peptides, and recombinant proteins**		

Dulbecco’s modified Eagle’s medium (DMEM), high glucose, pyruvate	Thermo Fisher Scientific	Cat# 41966029
Fetal bovine serum	Sigma-Aldrich (Merck)	Cat# S0615-500ML
Hygromycin B	Thermo Fisher Scientific	Cat# 10687010
Geneticin (G418 sulfate)	Thermo Fisher Scientific	Cat# 10131035
Puromycin dihydrochloride from *Streptomyces alboniger*	Sigma-Aldrich (Merck)	Cat# P7255-25MG
Doxycycline hyclate	Sigma-Aldrich (Merck)	Cat# D9891
Sodium butyrate	Sigma-Aldrich (Merck)	Cat# B5887
Potassium chloride	Sigma-Aldrich (Merck)	Cat# 3911
Disodium hydrogen phosphate	Sigma-Aldrich (Merck)	Cat# S3264
Potassium dihydrogen phosphate	Sigma-Aldrich (Merck)	Cat# P0662
Sodium chloride	Sigma-Aldrich (Merck)	Cat# 71380
Dako mounting medium	Agilent	Cat# CS70330-2
DAPI (4,6-diamidino-2-phenylindole dilactate)	Thermo Fisher Scientific	Cat# D3571
L-glutamine	Thermo Fisher Scientific	Cat# 25030081
Penicillin-streptomycin	Thermo Fisher Scientific	Cat# 15140122
MitoTracker Deep Red FM	Thermo Fisher Scientific	Cat# M22425
DMSO	Sigma-Aldrich (Merck)	Cat# D8418
Cacodylic acid, sodium salt, trihydrate	Sigma-Aldrich (Merck)	Cat# 205541
Calcium chloride dihydrate	Sigma-Aldrich (Merck)	Cat# 223506
Hydrochloric acid, 1 M	Sigma-Aldrich (Merck)	Cat# H9892
Formaldehyde, 16% w/v, methanol free, Ultra Pure	Polysciences	Cat# 18814-10
Glutaraldehyde, 50% w/v, grade I	Sigma-Aldrich (Merck)	Cat# G7651
Potassium ferrocyanide trihydrate	Sigma-Aldrich (Merck)	Cat# 455989
Osmium tetroxide 4% aqueous solution	Polysciences	Cat# 0972A-20
Osmium tetroxide 2% aqueous solution	Polysciences	Cat# 23310-10
Thiocarbohydrazide	Sigma-Aldrich (Merck)	Cat# 223220
Uranyl acetate dihydrate (discontinued)	Fluka (now part of Sigma-Aldrich/Merck)	Cat# 73943
Ethanol, absolute for analysis EMSURE	Sigma-Aldrich (Merck)	Cat# 100983
Epoxy embedding medium kit	Sigma-Aldrich (Merck)	Cat# 45359-1EA-F

**Experimental models: Cell lines**		

BAC16 bacmid iSLK cells	Provided by Prof. Jae Jung^5^	N/A
U2OS cells	ATCC; provided by Prof. Harald Wodrich	HTB-96
GFP-LC3B transduced U2OS cells	ATCC; generated by Katarina W. Schmidt^1^	HTB-96

**Software and algorithms**		

Fiji-ImageJ	Schindelin et al.^6^	http://fiji.sc; RRID: SCR_002285
Amira 3D software suite (v.2024.1)	Thermo Fisher Scientific	https://www.thermofisher.com/ch/en/home/electron-microscopy/products/software-em-3d-vis/amira-software.html; RRID: SCR_007353
Auto Slice & View 4 software for automated stack acquisition (v.4.2)	Thermo Fisher Scientific	https://www.thermofisher.com/order/catalog/product/AUTOSLICEVIEW4

**Other**		

1.2 μm syringe filters, sterile	Whatman	Cat# 10462261
Ultracentrifuge	Sorvall	RC6+
Ø 12mm round glass coverslips (0.17 mm thick)	HUBERLAB	Cat# 10.0360.52
24-well plates, flat bottom	VWR	Cat# 7002057
Glass-bottom culture dish, 35 mm, no. 1.5 gridded coverslip, 14 mm glass diameter (critical)	MatTek	Cat# P35G-1.5-14-C-GRD
Confocal laser scanning microscope	Leica Microsystems	SP8
PELCO BioWave Pro+ microwave processing system	Ted Pella	Cat# 36700-230
PELCO ColdSpot Pro	Ted Pella	Cat# 36116-10
PELCO EM Pro vacuum chamber	Ted Pella	Cat# 3536
Feather blades, double edge	Ted Pella	Cat# 121-9
SEM pin stubs, high-purity aluminum, 12.5 mm	Agar Scientific	Cat# AGG301P
EPO-TEK H20S adhesive	Electron Microscopy Sciences	Cat# 12672-20S
Vacuum coating system	Safematic GmbH	CCU-010 HV
Focused ion beam scanning electron microscope	Thermo Fisher Scientific	Helios 5 UX DualBeam

***Note:*** Sigma-Aldrich is part of Merck KGaA, Darmstadt, Germany. In North America, they operate under the name MilliporeSigma.

## Materials and equipment

**Table:** 0.3 M sodium cacodylate buffer with 4 mM CaCl_2_

Reagent	Final concentration	Amount
Cacodylic acid, sodium salt, trihydrate	0.3 M	64.209 g
Calcium chloride dihydrate	4 mM	0.5881 g
ddH_2_O	N/A	1 L
1 M Hydrochloric acid	N/A	ad pH 7.4
**Total**	**N/A**	**1 L**

Store at 4°C up to 1 year.

**CRITICAL:** Sodium cacodylate contains arsenic, making it toxic and potentially hazardous. Wear a lab coat and gloves. Must be handled in a fume hood.

**Table:** 0.25% glutaraldehyde + 2% formaldehyde solution

Reagent	Final concentration	Amount
16% w/v Formaldehyde Ultra Pure	2%	1.25 mL
50% w/v Glutaraldehyde	0.25%	0.05 mL
0.3 M Na-cacodylate buffer with 4 mM CaCl_2_	0.15 M	5 mL
ddH_2_O	N/A	3.7 mL
**Total**	**N/A**	**10 mL**

Prepare just before use.

**CRITICAL:** Aldehyde fixatives are hazardous. Wear a lab coat and gloves. Must be handled in a fume hood.

**Table:** 2.5% glutaraldehyde + 2% formaldehyde solution

Reagent	Final concentration	Amount
16% w/v Formaldehyde Ultra Pure	2%	1.25 mL
50% w/v Glutaraldehyde	2.5%	0.5 mL
0.3 M Na-cacodylate buffer with 4 mM CaCl_2_	0.15 M	5 mL
ddH_2_O	N/A	3.25 mL
**Total**	**N/A**	**10 mL**

Prepare just before use.

**CRITICAL:** Aldehyde fixatives are hazardous. Wear a lab coat and gloves. Must be handled in a fume hood.

**Table:** 3% potassium ferrocyanide + 4 mM calcium chloride

Reagent	Final concentration	Amount
Potassium ferrocyanide (K_4_Fe(CN)_6_)	3%	3 g
0.3 M Na-cacodylate buffer with 4 mM CaCl_2_	N/A	100 mL
**Total**	**N/A**	**100 mL**

Store at 4°C and protected from light up to 1 year.

**CRITICAL:** Potassium ferrocyanide becomes hazardous in acidic environments. Avoid contact with acids. Wear a lab coat and gloves. Must be handled in a fume hood.

**Table:** 2% reduced osmium

Reagent	Final concentration	Amount
Osmium tetroxide 4% aqueous solution	2%	2 mL
3% potassium ferrocyanide + 4 mM CaCl_2_	1.5%	2 mL
**Total**	**N/A**	**4 mL**

Prepare just before use.

**CRITICAL:** Osmium tetroxide is hazardous. Wear a lab coat and gloves. Must be handled in a fume hood.

**Table:** 1% thiocarbohydrazide

Reagent	Final concentration	Amount
Thiocarbohydrazide	1%	0.1 g
ddH_2_O	N/A	10 mL
**Total**	**N/A**	**10 mL**

Prepare just before use. Weigh in a glass vial and add ddH_2_O. Heat to 60°C, swirling the vial every 10 min to aid dissolution. Incubate solution at 60°C for at least 1 h. Keep warm until use. Just before use, filter through a 0.22 μm syringe filter.

**CRITICAL:** Thiocarbohydrazide is hazardous and potentially carcinogenic. Wear a lab coat and gloves. Must be handled in a fume hood.

**Table:** 1% uranyl acetate

Reagent	Final concentration	Amount
Uranyl acetate	1%	0.5 g
ddH_2_O	N/A	50 mL
**Total**	**N/A**	**50 mL**

Prepare the mixture in a 50 mL conical flask or wide-mouth bottle with a lid. Cover the bottle with aluminum foil to protect it from light exposure. Stir the mixture until uranyl acetate is fully dissolved. Store in a bottle or divide into aliquots at 4°C. Before use, filter through a filter with a pore size of 0.22 μm. Discard the solution if it becomes cloudy.

**CRITICAL:** Uranyl acetate is hazardous and radioactive. Wear a lab coat and gloves. Solid uranyl acetate must be handled in a fume hood. Avoid contaminating the lab space, and follow applicable regulations for handling, storing and discarding.

**Table:** 100% dry ethanol

Reagent	Final concentration	Amount
Ethanol, absolute for analysis	N/A	1 L
Molecular sieve, pore size ∼4 nm, in dialysis tubing	N/A	N/A
**Total**	**N/A**	**1 L**

Wash molecular sieve in ethanol until the solution is clear. Load molecular sieve submerged in ethanol into dialysis tubes (approx. 10 cm long), pre-soaked in ethanol, and close ends with plastic clips (dialysis tubing closure). Add the closed tube to a freshly opened bottle of ethanol. Refill the bottle with fresh ethanol once, then discard molecular sieve. Store at 20°C–22°C and in a fire-safe cabinet.

**Table:** Epoxy embedding medium/epoxy resin

Reagent	Final concentration	Amount
Epoxy Embedding Medium		14.5 g
Hardener MNA		10.5 g
Hardener DDSA		5.0 g
Accelerator DMP 30		0.54 g
**Total**	**N/A**	**∼24 mL**

Prepare just before use. Weigh epoxy embedding medium MNA and DDSA into a 50 mL centrifuge tube or disposable plastic beaker. Mix gently, but thoroughly for 10–15 min on a rotator or laboratory shaker. Avoid strong agitation and formation of air bubbles (these can be removed by applying a vacuum). Then add the accelerator and mix again for 10–15 min. Ready to use when the color of the mixture becomes uniform.

**CRITICAL:** Resin components are hazardous. Wear a lab coat and gloves. Must be handled in a fume hood.

## Step-by-step method details

### Identifying target cells and confocal imaging

**Timing: 4–8 h**

This section describes the labeling of mitochondria and confocal imaging to identify cells of interest, document their position, and record 3D images. Labeling mitochondria is critical as they are used as internal fiducial markers for the correlation of LM and EM datasets.

***Note:*** Unless otherwise indicated, use 1.5–2 mL of respective solution per culture dish for all further steps.

1. Labeling mitochondria with MitoTracker Deep Red.
  a. Prepare MitoTracker Deep Red staining solution.
    i. Prepare stock solution in DMSO according to manufacturer protocol (https://assets.thermofisher.com/TFS-Assets/LSG/manuals/mp07510.pdf).
    ii. Dilute stock solution to a final concentration of 66.6 nM (1:15000) in DMEM growth medium.
  b. Gently aspirate growth medium from the dish containing the KSHV-infected GFP-LC3B transduced U2OS and replace with staining solution.
  c. Stain the U2OS cells for no more than 5 min under growth conditions. The mitochondria take up the MitoTracker rapidly and can easily become over-stained.

***Note:*** We use MitoTracker Deep Red because its emission spectrum does not overlap with those of GFP or mCherry. Depending on the combination of fluorescent labels used, a different MitoTracker variant may be more appropriate.

**CRITICAL:** Make sure to prepare the MitoTracker Deep Red staining solution under a laminar flow hood to preserve sterile conditions. Keep the staining solution protected from light to avoid bleaching.

2. Fix cells in 0.25% glutaraldehyde and 2% formaldehyde in 0.15 M Na-cacodylate buffer, supplemented with 2 mM CaCl_2_.
  a. Gently aspirate staining solution from dish and replace with fixation buffer.
  b. Seal the dish with sealing film to protect the person handling samples from the glutaraldehyde toxicity during transport and LM imaging.
  c. Keep samples either at 20°C–22°C, or at 4°C and protected from light until confocal imaging.

***Note:*** Glutaraldehyde can cause autofluorescence. Use a low concentration if fixing cells prior to confocal imaging. Aldehyde-fixed cells can be stored in the described fixation buffer for up to several days. However, prolonged exposure may lead to fluorescence quenching or cell shrinkage. Alternatively, replace fixation buffer with pure Na-cacodylate or phosphate buffer for imaging. In this case, ensure a minimum fixation time of 15 min for cell monolayers, or use microwave-assisted fixation (see sample preparation for FIB-SEM section), before replacing fixative with buffer.

3. Confocal imaging: Identify a cell of interest, record a tile scan to document the position on the alphanumerical grid, and acquire a Z-stack.
  a. Use an inverted setup confocal microscope with a motorized XY stage and position saving functionality (we used a Leica SP8).
  b. Mount the sample dish on a stage insert which can hold culture dishes.
  c. Apply immersion oil.
  d. Identify the cell of interest showing double labeling for mScarletH:ORF65.KSHV and GFP-LC3B using a low magnification objective (in this case a 20× multi-immersion objective lens; HC PL APO CS2, NA 0.75).
  e. Store the XY position coordinates. This will allow the recovery of the cell position when moving the stage.
  f. Record a tile scan in transmission mode with the target cell in the center.
    i. Cover a total field of view that contains at least one full square of the grid (in this case 2 × 2 images).
    ii. Verify the letters and numerals can be identified to ensure relocation of the ROI in subsequent FIB-SEM imaging (see troubleshooting 1).
  g. Switch to a higher magnification objective lens (in this case a 63× oil immersion lens, HCX PL APO CS2, NA 1.4).
  h. Acquire a Z-stack of the target cell. In this example we recorded a Z-stack at a pixel size of 197 nm and Z-spacing automatically optimized by the Leica acquisition software which applies the Nyquist sampling criterion to determine the optimal step size.
  i. Excite GFP, mScarlet, and MitoTracker Deep Red sequentially using 488 nm, 555 nm, and 647 nm lasers at 5%–7% laser power, with fluorescence emission detected on HyD detectors using sequential scanning in the ranges of 500–550 nm for GFP, 571–610 nm for mScarlet, and 650–700 nm for MitoTracker Deep Red, while applying 2× line averaging and setting the voltage gain to 100%.

***Note:*** When recording more than one target cell per square of the alphanumerical grid, allow a minimum spacing of 500 μm between cells to enable FIB-SEM milling and stack acquisition without risking damage to neighboring ROI. We recommend selecting and documenting 3–5 ROIs prior to EM preparation, as not all may survive processing or yield optimal results.

**Pause point:** Aldehyde-fixed cells can be stored in Na-cacodylate buffer at 4°C for several days.

### Sample preparation for FIB-SEM

**Timing: 2–3 days**

**Timing: 6–7 h (steps 4–18)**

Aldehyde-fixed samples are post-fixed, stained, and resin-embedded for FIB-SEM using a microwave-assisted protocol. Microwave irradiation enhances reagent penetration and speeds up the preparation process.^7^ It is used throughout all steps up to resin infiltration with resin-ethanol mixtures (Steps 5–17). Complete resin infiltration can also be effectively carried out without using a microwave. Sample preparation can still be completed without a microwave, although this approach takes longer (∼2 days in total).

***Note:*** All microwave-assisted steps are performed in the PELCO BioWave Pro+ microwave processing system equipped with a ColdSpot Pro to maintain sample temperature. Where indicated, the PELCO EM Pro Vacuum Chamber is used to aid the penetration of solutions into the cells. The step-by-step microwave program, including vacuum settings and incubation times, is provided in the supplementary material.

**CRITICAL:** All the steps below should be performed in a fume hood, including the microwave-assisted steps. When using the vacuum chamber, remove lids from culture dishes.

4. Prepare all the solutions required for Steps 5–13, from 2.5% glutaraldehyde + 2% formaldehyde through to 1% uranyl acetate, as listed in the materials and equipment section in the order of use.
  a. Unless otherwise specified, prepare approximately 1.5–2 mL of each solution per culture dish.

***Note:*** Some solutions must be prepared just before use as indicated in the materials and equipment section.

5. Replace fixative buffer with fresh 2.5% glutaraldehyde and 2% formaldehyde in 0.15 M Na-cacodylate buffer, supplemented with 2 mM CaCl_2_.
  a. Put the culture dish (without lid) into vacuum chamber and post-fix samples using microwave on/off cycles (see Steps 1–4 of the microwave program in Table S1).
  b. Add ice into the vacuum chamber and carefully place the culture dish on it for the steps performed under high-wattage (650 W) microwave irradiation (Steps 5–7 in Table S1).
6. Rinse the cells three times in 0.15 M Na-cacodylate buffer with 2 mM CaCl_2_ (Steps 8–10 in Table S1).
7. Post-fix the cells with 2% reduced osmium.
  a. Exchange cacodylic buffer with 2% reduced OsO_4_.
  b. Treat the samples in the microwave with on/off cycles using the vacuum chamber (Steps 11–15 in Table S1).
8. Rinse the cells three times in ddH_2_O (Steps 16–18 in Table S1).

**CRITICAL:** Rinse thoroughly as residual OsO_4_ reacts with thiocarbohydrazide.

9. Incubate sample in thiocarbohydrazide.
  a. Load 1% thiocarbohydrazide solution into a syringe fitted with a 0.22 μm filter.
  b. Remove the ddH_2_O from the culture dish and add the filtered thiocarbohydrazide.
  c. Incubate the cells using microwave on/off cycles under vacuum (Steps 19–23 in Table S1).
10. Rinse the cells thoroughly in double distilled water.
  a. Rinse with ddH_2_O in the fume hood until solution becomes clear.
  b. Wash three times with ddH_2_O in the microwave (Steps 24–26 in Table S1).

**CRITICAL:** Residual thiocarbohydrazide can form a surface film on the solution. To remove this film, place the culture dish at an angle and gently flow ddH_2_O through the dish until the solution becomes clear.

11. Incubate cells with fresh 2% OsO_4_ using microwave on/off cycles under vacuum (Steps 27–31 in Table S1).
12. Rinse three times in ddH_2_O (Steps 32–34 in Table S1).
13. Treat cells with 1% uranyl acetate using microwave on/off cycles under vacuum (Steps 35–39 in Table S1).
14. Rinse three times in ddH_2_O (Steps 40–42 in Table S1).

***Note:*** Collect liquid waste from all washing steps following uranyl acetate staining and discard together with uranyl acetate solution.

**Pause point:** Samples can be stored in uranyl acetate at 4°C for up to 1 day. However, it is recommended to complete the preparation as soon as possible to avoid damage.

15. Dehydrate the cells using a series of ethanol solutions with increasing concentrations.
  a. Prepare 25, 50, 70 and 90% ethanol in ddH_2_O.
  b. Place the culture dish on ice while incubating in each ethanol solution using microwave on/off cycles under vacuum (Steps 43–54 in Table S1).
  c. Incubate three times in 100% dry ethanol, on ice, using microwave on/off cycles under vacuum (Steps 55–63 in Table S1).

***Note:*** Use only ethanol with polyethylene culture dishes. Other solvents may react with and degrade or damage the polyethylene.

16. Prepare epoxy embedding resin as described in materials and equipment section.

***Note:*** Epoxy resin mixture can be prepared during steps 13–15.

17. Infiltrate the cells with epoxy resin.
  a. Prepare mixtures of 25, 50 and 75% epoxy resin diluted with dry ethanol. Mix until the mixture reaches uniform consistency.
  b. Exchange ethanol with 25% epoxy resin diluted with ethanol.
  c. Process in the microwave in the vacuum chamber (Step 64 in Table S1).
  d. Gently aspirate the epoxy resin mixture, replace with fresh 25% epoxy resin and process in the microwave under vacuum (Step 65 in Table S1).
  e. Repeat steps (b-d), twice replacing with 50% epoxy resin diluted with ethanol, followed by two exchanges with 75% epoxy resin diluted with ethanol (Steps 66–69 in Table S1).

***Note:*** The viscosity of the resin mixture increases with increasing concentration of epoxy resin. Position culture dish at an angle and let the resin mixture drain, then pipette it off from the bottom of the tilted dish.

**Figure 1:**
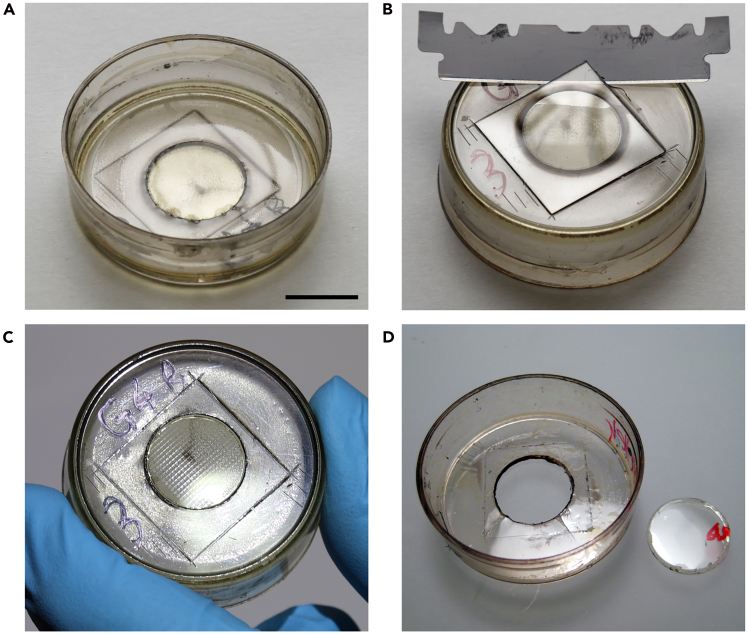
Resin embedding in a glass-bottom culture dish (A) Only the indent of the culture dish is filled with epoxy resin for polymerization. (B) The glass coverslip is detached from the polymerized resin using a thin razor blade. (C) After detachment, the alphanumerical grid remains visible as a topographical feature on the resin surface. (D) The resin disk is removed from the culture dish. Image reprinted with permission from Lucas et al., 2017.^2^ Scale bar: 1 cm.
  g. Wipe the rim of the plastic dish with a swab or paper tissue to remove excess resin.

***Note:*** Gently heating the resin to 50°C–60°C for a few minutes reduces its viscosity and can help in its removal, however prolonged heating may induce polymerization and should be avoided.

18. Infiltrate with 100% epoxy resin.
  a. Carefully pipette off 75% epoxy resin mixture and replace it with 100% epoxy resin.
  b. Place the dish on a laboratory shaker or rocker in the fume hood to ensure gentle agitation during infiltration.
  c. Incubate the cells for 1 h.
  d. Replace the epoxy resin. Drain the dish thoroughly to remove old resin, then incubate again for 1 h.
  e. Repeat step (d) for a total of 3–4 times, using fresh epoxy resin for each exchange.
  f. For the final resin exchange, fill only the indentation formed by the hole in the plastic dish, which is sealed by the cover glass bottom (Figure 1A).

**CRITICAL:** Do not overfill the indentation and make sure there are no bubbles. Carefully detach air bubbles sticking to the glass bottom of the dish with an eye lash glued to a tooth pick, or similar tool. Persistent air bubbles can be removed by vacuum.

19. Polymerize the resin in an oven at 60°C for 48 h.

### Mounting the resin-embedded cells for FIB-SEM

**Timing: ∼3–4 h**

**Timing: ∼15 min (per dish) (steps 20–21)**

**Timing: ∼3 h (steps 22–23)**

The cover glass at the bottom of the culture dish is removed from the polymerized resin disk. The resin disk is then extracted from the culture dish, mounted on an SEM stub, and sputter-coated to create a conductive surface for FIB-SEM analysis.

***Note:*** Removal of the cover glass, i.e. the growth substrate, exposes the embedded cells from the basal side. In FIB-SEM images the cells will appear up-side down.

20. Remove the cover glass to expose the cells.
  a. Remove the sample from the oven.
  b. Turn the dish upside down.
  c. Using a (single edged) razor blade or scalpel, carefully cut along the edges of the glass coverslip glued to the bottom of the dish.
  d. Apply gentle pressure and watch the glass separate from the resin at the edges.
    ***Note:*** Separation of the glass from the polymerized epoxy causes optical fringes, so-called Newton’s rings. This is easily visible under binoculars with tilted lighting.
  e. To completely detach the glass carefully slide a thin double-edged blade (we recommend Feather blades, broken in halves) underneath a corner of the glass coverslip parallel to the dish bottom (Figure 1B).
  f. Gently lift one edge of the blade and watch the glass detach.
  g. Repeat all around the glass edge until the glass coverslip fully detaches.
    ***Note:*** Separation of the cover glass and polymerized epoxy is most effective when the sample is fresh from the oven and still warm. Process multiple dishes one at a time and keep the remaining samples in the oven until use. See also troubleshooting 2.
21. Carefully remove the resin disk from the plastic dish frame. Place the dish upside down and apply gentle pressure to the resin disk using a finger or a flat tool to detach it from the frame (Figure 1D).

**CRITICAL:** Avoid touching the patterned side of the resin disk with bare hands to maintain its cleanliness. Ensure no broken glass residues remain on the resin surface. To prevent injury and surface scratches, place a lint-free paper tissue between the sample and your finger or tool.

**Pause point:** Resin-embedded specimens can be stored at 20°C–24°C for several month, or years. In case of high humidity, consider storing the sample in a desiccator to prevent moisture-related issues.

***Note:*** We typically mount the entire resin disk for FIB-SEM, but it is also possible to mount just a portion of it. Carefully cutting the resin disks into smaller pieces allows for the extraction of specific areas, which can e.g. be remounted to prepare ultrathin (serial) sections for (S)TEM.

22. Mount the samples for FIB-SEM.
  a. Allow the sample to cool to 20°C–22°C.
  b. Prepare the EPO-TEK H20S adhesive by mixing equal amounts of components A and B.
  c. Apply adhesive to the SEM stub and mount epoxy disk with cells facing upward. Apply light pressure to ensure good contact.
  d. Paint the overhanging rim and the sides of the resin disk with adhesive to improve conductivity.
  e. Cure the adhesive at 80°C for 2 h.
  f. Allow the sample to cool to 20°C–22°C before proceeding.

***Note:*** EPO-TEK H20S cures faster at higher temperatures; however, this may negatively affect the resin structure. Conversely, curing at lower temperatures is possible but will require more time. Alternatively, silver or carbon conductive paint can be used to mount the resin disks for FIB-SEM.

23. Apply an 8 nm layer of platinum-palladium using a sputter coater (here a Safematic CCU-010) to enhance the specimen’s conductivity.

***Note:*** Alternative sputtering targets, such as platinum or gold, can also be used for coating.

**Pause point:** Mounted **s**amples can be stored under dry conditions at 20°C–24°C for several month, or years. In case of high humidity, consider storing the sample in a desiccator to prevent moisture-related issues.

### Relocating cells of interest and FIB-SEM stack acquisition

**Timing: approx. half a day for relocation of ROI and setting up automated FIB-SEM volume acquisition + 24–48 h for actual volume acquisition (per sample/ROI)**

This section outlines the procedure for relocating the cell of interest in the resin disk and setting up the image stack acquisition in FIB-SEM. Relocation is facilitated by the alphanumerical grid, which was etched into the glass substrate and is now visible as a topographical structure on the resin surface (Figure 1C), making it detectable in SE imaging. Having the LM data at hand helps to identify the cell of interest.

***Note:*** We used a Thermo Fisher Scientific Helios 5 UX Dual Beam, equipped with an InColumnBSD detector. The described milling strategies and imaging conditions refer specifically to this instrument and may need to be adapted for FIB-SEM systems from other manufacturers or models.

24. Prepare the LM image data.
  a. Prepare composite images to identify amphisomes where the mScarlet signal for ORF65 from recombinant KSHV and the GFP signal of the autophagosomal marker LC3B co-localize.
  b. Check the confocal image data and determine the approximate depth of the structure of interest. This helps to define the milling depth for FIB-SEM stack acquisition.
  c. For guidance to relocate the target cell, prepare z-projection images of confocal stacks and superimpose the transmission mode image of the alphanumerical grid to show the cell’s exact position on the grid using Fiji-ImageJ^6^ software.

***Note:*** LM images may be mirror-inverted compared to the representation of the sample in the FIB-SEM.

**Figure 2:**
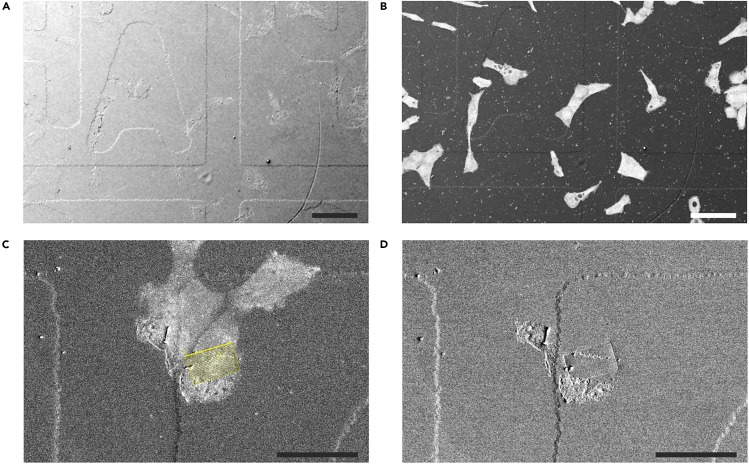
Relocating the ROI in the FIB-SEM (A) Low-voltage SE2 imaging (2 kV) of the resin surface shows the imprint of the alphanumerical finder grid. (B) Increasing the acceleration voltage to 18 kV expands the electron beam’s interaction volume with the sample, enabling the detection of both BSE and SE2 signals from within the resin block. This reveals cells stained with heavy metal salts, enabling precise FIB milling. Images reprinted and adapted with permission from Lucas et al., 2017.^2^ (C) Cell of interest imaged at 18 kV. The region for FIB-SEM stack acquisition is marked in yellow. (D) A shallow rectangular indent is milled around it, which remains detectable also at low acceleration voltage. Scale bars: (A and B) 100 μm, (C and D) 50 μm.
  c. Navigate to the square containing the cell of interest, as determined using LM in section “Identifying target cells and confocal imaging”.
  d. Increase the SEM acceleration voltage to 15–18 kV to visualize the heavy metal-stained cells within the resin (Figure 2B).
  e. Detect the cell of interest and mark the region for 3D imaging by milling a shallow rectangular indent around it (Figure 2C and 2D) to ensure it remains detectable even at low acceleration voltage, using an electron beam milling strategy at 18 kV and 1.6 nA, milling time 5 s.
  f. Reset the acceleration voltage to 2 kV, and imaging current of 0.1 nA.
    ***Note:*** A higher acceleration voltage increases the interaction volume of the electron beam with the specimen, allowing signals to be detected from heavy metal-stained cells beneath the resin surface.
    ***Note:*** Most FIB-SEM instruments offer navigation tools based on previously acquired images, such as the LM images.
27. Add protective layer deposition.
  a. Use the ion beam deposition to apply a protective carbon cap of approx. 1–2 μm thickness to protect the sample and minimize milling artifacts.
  b. Ensure the protective layer is large enough to cover the complete region of interest. In this study, we chose ROIs of approx. 20 × 15 μm^2^.

***Note:*** Other materials such as platinum can be used as protective layer.

25. Preparation for FIB-SEM.
  a. Load the sample into FIB-SEM.
  b. Adjust the working distance for both the SEM and FIB.
  c. Tilt the sample so the sample surface is perpendicular to the ion-beam (52° in this case) and align the electron and ion beams to ensure proper coincidence of the SEM and FIB focal points at the sample surface.
26. Relocate the cell of interest.
  ***Note:*** Document position of the target cell and imaging ROI, as well as the locations of the carbon deposition and FIB milling window before and after the stack acquisition with SEM images. This will help in aligning the confocal and FIB-SEM volume data.
  a. Set the SEM acceleration voltage to 2 kV to image the resin surface.
  b. Choose a slow scan speed and/ or high pixel integration to visualize the shallow finder grid structure on the resin surface (Figure 2A).

***Optional:*** Linear marks can be milled either into the protective layer or into the resin surface next to, but not on top of, the ROI. These can help validate the result of image registration (see step 30 – Postprocessing FIB-SEM data).

28. Expose the ROI for FIB-SEM milling.
  a. Mill a front trench to remove material in front of the ROI. Make the trench twice as wide as the ROI.
  b. Mill side trenches alongside the ROI. Choose the scan direction such that milling is directed towards the ROI.
  c. If available use a water-enhanced multipass milling strategy for silicon at 9.9 nA milling current.
    ***Note:*** Water-enhanced milling increases sputtering efficiency, i.e. milling speed.
  d. Polish the cross-section at lower milling current, e.g. 30 kV and 2.6 nA, with a milling strategy for silicon cross-sections to generate a smooth surface suitable for high-resolution imaging.
    ***Note:*** Side trenches minimize redeposition by serving as collection areas for sputtered material, preventing it from accumulating on the milled surface. They enable precise milling without distortions, making them especially useful for 3D reconstruction.
    ***Note:*** Protective layer deposition and trench milling can also be done with the Thermo Fisher Auto Slice & View wizard.
    **Pause point:** ROI definition, protective layer deposition, and trench milling can be completed in a single session. If necessary, the sample can then be unloaded from the FIB-SEM. Stack acquisition can be started in a separate session. However, it is recommended to let the FIB-SEM stabilize for a few hours or overnight after loading a specimen to ensure optimal stability.
29. Set up automated serial milling and imaging.
  ***Note:*** Most FIB-SEMs offer a wizard to set up automated stack acquisition, we used Thermo Fisher Auto Slice & View 4 software. The following steps describe the workflow for this software.
  a. Create a fiducial marker next to the ROI to enable a stable recognition of the milling edge and to compensate for drift using the automated wizard (1 μm carbon deposition, 2 μm milling depth for the fiducial shape, milling parameters 30 kV and 1.2 nA).
  b. Select either ETD or TLD SE detectors for FIB imaging.
  c. Choose a pixel size of approx. 10 nm, using a pixel count of 2000–3000 and a dwell time of 500 ns–1 μs.
    ***Note:*** Milling accuracy is approx. 1/5 of the imaging resolution, therefore choose the pixel size for FIB imaging accordingly, i.e. for cutting 10 nm slices, a pixel size of at least 50 nm is required.
  d. Optimize brightness and contrast to enable detection of the milling edge.
  e. Define the milling window approx. 5–10 μm wider than the ROI.
  f. Select the slice thickness (10 nm in our case), the milling depth (here 10 μm) and an ion beam current of 0.26 nA using a milling strategy for cross-sections on silicon.
  g. Specify the imaging window and optimize the SEM imaging parameters.
    i. Set acceleration voltage to 2 kV.
    ii. Choose an imaging current of 0.40–0.80 nA.
    iii. Select the InColumnBSD detector, or MD detector.
    iv. Use a dwell time of approx. 3 μs with a line averaging dwell strategy (line averaging = 4).
  h. Define the imaging pixel size such that it matches the smallest expected feature.
    ***Note:*** FIB-SEM can achieve an imaging pixel size of 4–5 nm. Smaller pixel sizes and thus a higher pixel count (if the field of view is not altered) will result in a prolonged acquisition time. Define an appropriate balance.
  i. Focus and correct the astigmatism. Use auto functions for focus, stigmation, and lens alignment during the stack acquisition.
  j. Set the tilt correction to −38°.
  k. Choose an appropriate shift correction strategy.
  l. Start the image stack acquisition and monitor the process: check image quality regularly and adjust settings if needed.

### 3D correlation of LM and EM data and 3D modeling

**Timing: 1–2 days per dataset**

FIB-SEM data is correlated with the confocal data to locate the target structure in the EM data. This last section outlines the post-processing steps for FIB-SEM data and the alignment process with confocal images using the Amira 3D software suite.

***Note:*** Various software tools, including combinations of multiple applications, can be used to accomplish the described tasks. For example, Fiji-ImageJ^6^ provides plugins for most processing and visualization steps, ecCLEM^8^ can be used to register the fluorescent images to the EM data, and several options such as Blender, Imaris, IMOD, or Microscopy Image Browser^9^ are available for 3D visualization and modeling.

30. Postprocess the FIB-SEM data.
  a. Load the FIB-SEM data in Amira and (if needed) compensate for potential fluctuations in brightness and contrast: calculate a *Background Image* and subtract it from the original image stack (Figure 3A).

**Figure 3:**
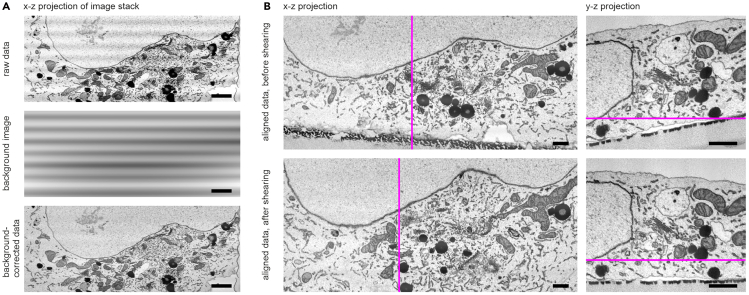
Postprocessing of the FIB-SEM data (A) The x-z projection of a FIB-SEM image stack may show fluctuations in brightness and contrast, appearing as horizontal stripe patterns in the raw data. To correct this, a background image was generated using the Amira software suite and subtracted from the original image stack, effectively removing the artifact. (B) Aligning the image stack often creates a tilted surface, most noticeable in the y-z projection (top right). Since the resin surface is known to be straight, this distortion can be corrected using a shear operation (bottom row). The magenta lines indicate the positions along which the orthographic projections were calculated. Scale bars: (A) 2 μm, (B) 1 μm.
  b. Register the image stack using the *Align Slices* module of Amira. Choose *Least-squares alignment mode* and do not allow rotation for the automatic alignment.
  c. Then resample the aligned image stack to create a new dataset with the alignment result.
  d. Correct the tilt of the resulting image stack using a *Shear* operation. Choose the shear angle such that the surface of resin block is straight (Figure 3B).
    ***Note:*** We prefer to use a rigid alignment model for stack alignment to avoid introducing additional distortions. However, it is important to note that any alignment process can introduce some degree of bias or distortion, depending on the data quality and the chosen parameters.
  e. Crop dataset to remove voxels which do not contain relevant data.
  f. Filter mildly, using e.g. a Gaussian or a Median filter, to reduce background noise.
31. Align the FIB-SEM data with confocal images.
  a. Load the confocal dataset, and available overview LM and SEM images showing the position and orientation of the ROI and FIB milling window with respect to the cell’s position on the finder grid.
  b. Align the overview images and confocal stack either by manually moving them with the mouse (using the *Translate tool*) or by entering specific positioning values in the *Transform Editor.* Use features of the alphanumerical grid which are visible in both image modalities as reference points for alignment.
  c. Make sure to match the orientation of confocal and FIB-SEM datasets, i.e. top and bottom are aligned.
  d. If needed, mirror the confocal data to match the orientation of the FIB-SEM data.
  e. Rotate the FIB-SEM data 90° around the X-axis.
    **CRITICAL:** While the image plane of the confocal data is parallel to the cell substrate (i.e., the resin surface), the milling and image plane of FIB-SEM are perpendicular to the resin surface. To correlate the two datasets, one of the image stacks must be rotated by 90°.
  f. In order to establish the correlation, move one of the two datasets, i.e. translate and rotate, with respect to the other. Use the cell substrate, visible in both imaging modalities, as the anchoring reference for the cell’s base.
    ***Note:*** We recommend reslicing both datasets virtually to visualize them in the same imaging plane.
  g. Check the correlation result using overlay functions, e.g. a *Color Wash* module in Amira, blending e.g. the FIB-SEM data onto the confocal data (Figure 4B).

**Figure 4:**
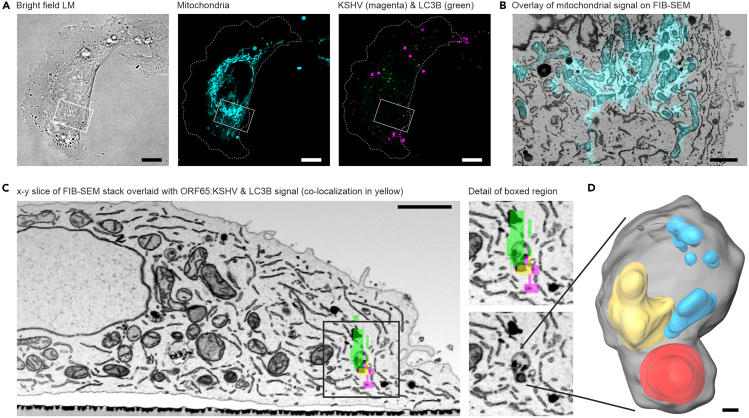
Fluorescence-guided FIB-SEM allows precise targeting of virus-containing amphisomes (A) Aldehyde-fixed cells were imaged with confocal microscopy to identify target cells. Amphisomes were identified by co-localization of mScarlet-labeled ORF65 from recombinant KSHV (magenta) with the autophagosomal marker LC3B-GFP (green), appearing as yellow. MitoTracker Deep Red (cyan) served as an internal fiducial marker for precise correlation with FIB-SEM data. (B) Overlay of MitoTracker Deep Red signal on an x-z projection of the correlative FIB-SEM data. (C) Overlaying co-localized mScarlet:ORF65:KSHV and LC3B-GFP signals onto the FIB-SEM image stack identifies the precise location of an amphisome. The enlarged detail (corresponding to the boxed region in the left image) shows a cross-section of an amphisome with a virus particle at the bottom. A 3D reconstruction of this amphisome is shown in (D), with KSHV in red, lipid-rich structures in yellow, and intraluminal structures in blue. Images reprinted and adapted with permission from Schmidt et al., 2024.^1^ Scale bars: (A) 10 μm, (B) 2 μm, (C) 2 μm, (D) 50 nm.
  h. Use the representation of mitochondria in the confocal and FIB-SEM images as fiducials to fine-tune the correlation. Judge the quality of the alignment by visually inspecting how well distinct mitochondrial structures overlap across the two modalities. Consistent shape, position, and orientation serve as good indicators of accurate correlation.
    ***Note:*** The shape and position of the nucleus can also be helpful for fine-tuning the correlation. Even if the nucleus was not specifically labeled for LM, it may be visible in transmission LM, or appear as a negative shape e.g. when using cytosolic fluorescent labels.
  i. If the correlation does not align properly see troubleshooting 3 section.
  j. Once the correlation is established, identify the target structure in the FIB-SEM data using the overlay of the respective fluorescent signal, in our case the co-localization of KSHV capsid tagged with mScarlet with the GFP-LC3B autophagy marker (Figure 4C).
32. If of interest, segment the target structure, in this case the virus-containing vesicles and their intraluminal structures, and represent the segmentation results in 3D (Figure 4D).
  ***Note:*** Segmentation can be performed using a variety of approaches and tools and software. The workflow we describe here serves as one example and is not intended to be exhaustive.
  a. In the Amira *Segmentation Editor*, create a *Label Image* and name it to reflect the structure to be segmented (e.g., “vesicle”). By default, a *Label Image* contains two materials to which voxels can be assigned: “Exterior” and “Material 1.”
  b. Mask the voxels in all consecutive image planes that depict the target structure using an appropriate segmentation tool (e.g., the *Brush* tool for manual segmentation).
  c. Assign the masked voxels to “Material 1” of the *Label Image.*
  d. Repeat steps (a-c) for each additional structure of interest.
  e. For visualization return to the Amira *Project View* and attach a *Generate Surface* module to the *Label Image* icon.
  f. Select settings appropriate for your visualization task (e.g. smoothing model and extent, closing the structure at borders). In our example we used unconstrained smoothing, extent 5, and closed borders. Then start the surface generation.
  g. Attach a *Surface View* module to the resulting data icon.
  h. In this example we segmented the amphisome membrane, the enclosed KSHV particles, lipid-rich structures, and other intraluminal structures. To enhance visibility of the internal elements, the amphisome membrane was rendered transparent using the *Surface View* module.
  i. Use the *Snapshot* tool to export still images of the segmented 3D structure.

## Expected outcomes

The presented workflow for volumetric CLEM enables the targeting of rare cellular events, such as virus-containing amphisomes. Key features include the use of gridded glass bottom dishes, which facilitate the relocation of the target cell in FIB-SEM, and the use of mitochondria as internal fiducial landmarks for correlation (Figure 4A and 4B). This approach allows the use of established LM assays, both for live cell or fixed cell imaging, to identify events or structures of interest.

This LM-guided approach makes high-resolution FIB-SEM more efficient and reduces acquisition time. The resulting high-resolution, multi-scale volume data combines functional information obtained by confocal fluorescence microscopy with ultrastructural details provided by FIB-SEM. Acquiring isotropic voxels in FIB-SEM allows precise 3D modeling of the target structure. Besides providing visualization of the three-dimensional organization of, for example, organelle structure or content (Figure 4D), this enables statistical analyses, e.g. volume or surface measurements.

In our case, this protocol allowed precise identification of virus-containing amphisomes, which were key to understanding the role of macroautophagy in regulating KSHV entry. The 3D reconstruction of vesicles in which mScarletH:ORF65.KSHV and GFP-LC3B co-localized revealed intact KSHV virions, suggesting that autophagosome recruitment does not require viral envelope fusion with endosomal membranes. Indeed, the interaction of the KSHV envelope glycoprotein gH with its receptor EphA2 was found to be required for autophagosome recruitment to KSHV-containing endosomes.^1^

## Limitations

The described workflow is designed to optimize imaging conditions for both microscopy modalities involved. However, several aspects of this staggered approach can influence the results.

Chemical fixation is known to induce artifacts such as shrinkage, swelling, or distortion. It is also relatively slow, making it difficult - if not impossible - to capture fast, transient cellular events. While in cell monolayers fixation at the outer cell membrane may occur instantaneously, it can take up to 4 min for the fixative to fully penetrate the cell.^10^ To minimize artifacts, the concentration, composition, osmolarity, and pH of the fixation buffer should be carefully adjusted and optimized. Glutaraldehyde can induce autofluorescence. And while formaldehyde penetrates specimens faster than glutaraldehyde, its reactions with proteins are reversible and it can be removed by washing with water. Additionally, the subsequent steps of sample preparation for EM, including staining, dehydration, and resin embedding can alter cell shape and ultrastructure. This may cause difficulties with correlation and negatively influence the relocation and identification precision of target structures.

Physical fixation, i.e. cryo-immobilization, offers an alternative which can prevent these artifacts and allows fixation within milliseconds. This makes it particularly useful for capturing rapid cellular events. However, the required experimental setup and instrumentation are more complex, requiring significant financial investment and specialized expertise.

So-called in-resin fluorescence approaches offer an alternative to overcome issues related to sample deformation during preparation. These can be applied following both, chemical and physical fixation and enable the combination of LM and EM on a single, embedded specimen, rather than relying on separate prepartations.^11^

The described approach relies on manual alignment of the confocal and FIB-SEM images. The correlation precision is therefore operator-dependent. The resolution mismatch of confocal and FIB-SEM imaging along the Z-axis of confocal data can be challenging. However, warping the data to force a better correlation should be avoided, as it may lead to incorrect conclusions about the localization of the target structures. In this case, the introduction of additional fiducial markers,^12^ although they too can cause artifacts, can help improve correlation precision.

## Troubleshooting

### Problem 1

Letters and numerals of the coverslip grid cannot be identified in confocal transmission images.

### Potential solution

The use of immersion liquids for confocal imaging can obscure the visibility of the finder grid. Clean the glass bottom and (re-)image using an air objective lens. To avoid shifting the culture dish and loosing track of the correct position, it can be helpful to fix the dish on the stage e.g. with a reusable, adhesive putty.

**CRITICAL:** Start imaging with air objective lenses and make sure to acquire all required images with this lens, before switching to an oil immersion objective lens. Switching back from oil immersion to air objectives is not advisable.

### Problem 2

Glass coverslip does not detach, or breaks into pieces.

### Potential solution

The glass detaches best, when glass and resin are fresh from the polymerization oven, i.e. still warm. Ensure that the resin is well polymerized. Warm it again in the polymerization oven, then try again. If the glass breaks into pieces that cannot be removed without risking to damage the resin surface, briefly dip the warm resin into liquid nitrogen until the glass detaches. Then lift off glass fragments carefully with tweezers.

If any glass fragments remain and cannot be removed, consider cutting out the region containing the target cell(s) using a jeweler’s saw or sharp razor blade. If the remaining glass covers the ROI, hydrofluoric acid (HF) can be applied to remove it. However, HF is extremely dangerous and must be handled with great care. It is both highly corrosive and toxic, so ensure that appropriate safety measures and an emergency protocol are in place.

### Problem 3

The confocal and FIB-SEM data do not match due to distortion.

### Potential solution

The cell shape can be altered during EM sample preparation. Additionally, the registration of the FIB-SEM image stack can induce distortions, such as shear. Both can result in a mismatch. If fiducial landmarks can be clearly identified in both image modalities, it can be considered to warp the FIB-SEM dataset to match the confocal images. Landmark-based warping can e.g. be performed using the Amira 3D software suite as described by Karreman et al.^13^ However, warping should be used cautiously.

## Resource availability

### Lead contact

Further information and requests for resources and reagents should be directed to and will be fulfilled by the lead contact, Miriam S. Lucas (miriam.lucas@scopem.ethz.ch).

### Technical contact

Technical questions on executing this protocol should be directed to and will be answered by the technical contacts: Miriam S. Lucas (miriam.lucas@scopem.ethz.ch), who is the contact for correlative imaging, and Christian Münz (muenzc@immunology.uzh.ch), who is the contact for cell culture, virus production, and virus infection.

### Materials availability

This study did not generate new unique reagents.

### Data and code availability

No additional data was used. This paper does not report original code. Any additional information for reanalyzing this work is available from the lead contacts upon request.

## Acknowledgments

Confocal imaging was done at the Center for Microscopy and Image Analysis, Zurich. This research was supported by the Swiss National Science Foundation (310030_204470/1, 310030L_197952/1, and CRSII_222718_10000065), Cancer Research Switzerland (KFS-5896-08-2023-R), the Swiss MS Society (2023-17), the Swiss State Secretariat for Education, Research and Innovation (SERI) for EU Horizon BEHIND-MS, the Sobek Foundation, the Swiss Vaccine Research Institute, and Pfizer.

## Author contributions

Conceptualization and methodology, M.S.L.; investigation, M.S.L. and K.W.S.; validation, M.S.L. and C.M.; funding acquisition, C.M.; writing – review and editing, M.S.L. and C.M.

## Declaration of interests

The authors declare no competing interests.
