# Supplementary material for: Correlative volume microcopy of virus-containing amphisomes enabled by fluorescence-guided focused ion beam-scanning electron microscopy
Source: STAR Protoc. 2025 Aug 15;6(3):104020. doi: 10.1016/j.xpro.2025.104020 (PMC12392765; doi:10.1016/j.xpro.2025.104020)
Supplement: Document S1. Table S1 [file mmc1.pdf]

Table S1: Microwave program for PELCO BioWave® Pro+ sample processing system, related to Steps 4-17.

Step #	Description	Time [min]	Time [sec]	Power [W]	Vacuum cycle [1=auto, 0=off]	Vacuum set point	Steady Temp temp. [°C]	Max. temp. [°C]	Load Cooler Set Point [°C]	User prompt [1=yes, 0=no]
1	Fixative solution	1	30	100	1	20	5	10	15	1
2	Fixative solution	1	30	0	1	20	5	10	15	0
3	Fixative solution	1	30	100	1	20	5	10	15	0
4	Fixative solution	1	30	0	1	20	5	10	15	0
5	Fixative solution, on ice	0	10	650	1	20	5	10	15	1
6	Fixative solution, on ice	0	20	0	1	20	5	10	15	0
7	Fixative solution, on ice	0	10	650	1	20	5	10	15	0
8	Na-cacodylate buffer	0	40	100	0	20	5	20	15	1
9	Na-cacodylate buffer	0	40	100	0	20	5	20	15	1
10	Na-cacodylate buffer	0	40	100	0	20	5	20	15	1
11	2% OsO_4 + 1.5% $K_3Fe(CN)_6$ + 2 mM $CaCl_2$	2	0	100	1	20	5	20	15	1
12	2% OsO_4 + 1.5% $K_3Fe(CN)_6$ + 2 mM $CaCl_2$	2	0	0	1	20	5	20	15	0
13	2% OsO_4 + 1.5% $K_3Fe(CN)_6$ + 2 mM $CaCl_2$	2	0	100	1	20	5	20	15	0
14	2% OsO_4 + 1.5% $K_3Fe(CN)_6$ + 2 mM $CaCl_2$	2	0	0	1	20	5	20	15	0
15	2% OsO_4 + 1.5% $K_3Fe(CN)_6$ + 2 mM $CaCl_2$	2	0	100	1	20	5	20	15	0
16	ddH_2O	0	40	100	0	20	5	20	15	1
17	ddH_2O	0	40	100	0	20	5	20	15	1
18	ddH_2O	0	40	100	0	20	5	20	15	1
19	1% Thiocarbohydrazide	1	0	100	1	20	5	20	15	1
20	1% Thiocarbohydrazide	1	0	0	1	20	5	20	15	0
21	1% Thiocarbohydrazide	1	0	100	1	20	5	20	15	0
22	1% Thiocarbohydrazide	1	0	0	1	20	5	20	15	0
23	1% Thiocarbohydrazide	1	0	100	1	20	5	20	15	0
24	ddH_2O	0	40	100	0	20	5	20	15	1
25	ddH_2O	0	40	100	0	20	5	20	15	1
26	ddH_2O	0	40	100	0	20	5	20	15	1
27	2% OsO_4	2	0	100	1	20	5	20	15	1
28	2% OsO_4	2	0	0	1	20	5	20	15	0
29	2% OsO_4	2	0	100	1	20	5	20	15	0
30	2% OsO_4	2	0	0	1	20	5	20	15	0
31	2% OsO_4	2	0	100	1	20	5	20	15	0
32	ddH_2O	0	40	100	0	20	5	20	15	1
33	ddH_2O	0	40	100	0	20	5	20	15	1
34	ddH_2O	0	40	100	0	20	5	20	15	1
35	1% Uranyl acetate	2	0	100	1	20	5	20	15	1
36	1% Uranyl acetate	2	0	0	1	20	5	20	15	0
37	1% Uranyl acetate	2	0	100	1	20	5	20	15	0
38	1% Uranyl acetate	2	0	0	1	20	5	20	15	0
39	1% Uranyl acetate	2	0	100	1	20	5	20	15	0
40	ddH_2O	0	40	100	0	20	5	20	15	1
41	ddH_2O	0	40	100	0	20	5	20	15	1
42	ddH_2O	0	40	100	0	20	5	20	15	1
43	25% Ethanol, on ice	1	0	150	0	20	5	20	15	1
44	25% Ethanol, on ice	1	0	0	0	20	5	20	15	0
45	25% Ethanol, on ice	1	0	150	0	20	5	20	15	0
46	50% Ethanol, on ice	1	0	150	0	20	5	20	15	1
47	50% Ethanol, on ice	1	0	0	0	20	5	20	15	0
48	50% Ethanol, on ice	1	0	150	0	20	5	20	15	0
49	75% Ethanol, on ice	1	0	150	0	20	5	20	15	1
50	75% Ethanol, on ice	1	0	0	0	20	5	20	15	0
51	75% Ethanol, on ice	1	0	150	0	20	5	20	15	0

52	90% Ethanol, on ice	1	0	150	0	20	5	20	15	1
53	90% Ethanol, on ice	1	0	0	0	20	5	20	15	0
54	90% Ethanol, on ice	1	0	150	0	20	5	20	15	0
55	Dry Ethanol, on ice	1	0	150	0	20	5	20	15	1
56	Dry Ethanol, on ice	1	0	0	0	20	5	20	15	0
57	Dry Ethanol, on ice	1	0	150	0	20	5	20	15	0
58	Dry Ethanol, on ice	1	0	150	0	20	5	20	15	1
59	Dry Ethanol, on ice	1	0	0	0	20	5	20	15	0
60	Dry Ethanol, on ice	1	0	150	0	20	5	20	15	0
61	Dry Ethanol, on ice	1	0	150	0	20	5	20	15	1
62	Dry Ethanol, on ice	1	0	0	0	20	5	20	15	0
63	Dry Ethanol, on ice	1	0	150	0	20	5	20	15	0
64	25% Epon in Ethanol	3	0	150	1	20	5	20	15	1
65	25% Epon in Ethanol	3	0	150	1	20	5	20	15	1
66	50% Epon in Ethanol	3	0	150	1	20	5	20	15	1
67	50% Epon in Ethanol	3	0	150	1	20	5	20	15	1
68	75% Epon in Ethanol	3	0	150	1	20	5	20	15	1
69	75% Epon in Ethanol	3	0	150	1	20	5	20	15	1
